# Epidemiology, treatment, and recurrence of odontogenic and non-odontogenic cysts in South Sulawesi, Indonesia: A 6-year retrospective study

**DOI:** 10.4317/jced.59309

**Published:** 2022-03-01

**Authors:** Muhammad Ruslin, Kiara N. van Trikt, Andi-Sitti-Hajrah Yusuf, Andi Tajrin, Abul Fauzi, Muhammad-Irfan Rasul, Paolo Boffano, Tymour Forouzanfar

**Affiliations:** 1 Department of Oral and Maxillofacial Surgery, Faculty of Dentistry, Hasanuddin University, Makassar, Indonesia; 2 Department of Oral and Maxillofacial Surgery/Oral Pathology, Amsterdam University Medical Centers and Academic Centre for Dentistry Amsterdam (ACTA), Vrije Universiteit Amsterdam, Amsterdam Movement Sciences, Amsterdam, The Netherlands; 3 Division of Maxillofacial Surgery, “Maggiore della Carità” University Hospital, University of Eastern Piedmont, Novara, Italy

## Abstract

**Background:**

Diagnosis of jaw cysts is challenging in general dental practice, and most cases are incidentally discovered through routine dental radiography. The aim of this study was to examine the epidemiology and treatment of odontogenic and non-odontogenic cysts to better understand the status of these lesions in populations in South Sulawesi, Indonesia.

**Material and Methods:**

This retrospective study was conducted on patients treated at four different hospitals in Makassar between January 2011 and June 2017. Patients diagnosed as having odontogenic or non-odontogenic cysts were included in the study. Information on variables such as sex, age, histopathological, and anatomical distributions was collected. Statistical analyses were performed using an independent T-test and the Pearson chi-square test (*p*< 0.05).

**Results:**

A total of 173 samples were collected, of which only 60 were histopathologically analyzed. The patients’ mean age was 30.3 years. The cysts occurred more frequently in women and in the anterior maxilla, followed by the posterior mandible. The radicular cyst was the most prevalent type, followed by the dentigerous cyst. Most cysts were treated with enucleation. Of the patients, 72.8% were followed up, of whom 3.2% had a recurrence and only 19.1% had complaints of clinical symptoms.

**Conclusions:**

Our findings indicate that odontogenic and non-odontogenic cysts widely vary in terms of incidence, with some exhibiting a predilection for specific ages and sites and specific sex. Knowledge of these factors could be useful for both clinicians and pathologists in the diagnosis and choice of the appropriate treatment plan.

** Key words:**Cyst, epidemiology, odontogenic, non-odontogenic, treatment.

## Introduction

Cysts of the jaw comprise an important aspect of oral maxillofacial (OMF) pathology, with an increasing number of cases being discovered incidentally through routine radiography in general dental practice (1). In the OMF pathology, cysts are classified into two groups, namely odontogenic and non-odontogenic cysts, which differ in cyst formation (2,3). According to Grossman *et al*., odontogenic and non-odontogenic cysts account for 0.8%–45.9% and 0.5%–1.01% of all specimens submitted to oral pathologists, respectively (4). In a systematic review, Johnson *et al*. revealed that odontogenic cysts occurred 2.25 times more frequently than odontogenic tumors (5). To date, several studies have reported about epidemiology of odontogenic and non-odontogenic cysts in various populations such as those of Brazil (4), Queensland (6), and the United Kingdom (7). However, to our knowledge, studies that mainly focus on the biological and histopathological behaviors of odontogenic and non-odontogenic cysts in the Indonesian population are sparse. In view of the lack of records about these lesions, conducting studies on this topic in Indonesia seems reasonable.

Knowledge of the biological characteristics, basic features, and histopathological behaviors of cystic lesions in the OMF region is key to ensuring an early clinical diagnosis, a prompt treatment, and a favorable prognosis (8-10). In most cases, diagnosis of odontogenic and non-odontogenic cysts can be challenging owing to their quiescent progression and similar clinical and radiographic characteristics (11-13). The asymptomatic nature of these cysts in the initial phase delays diagnosis and treatment and thereby leads to aggressive growth of the cysts and destruction of adjacent regions such as the maxillary sinus and mandibular nerve (14-16). These factors can also cause unfavorable aesthetic and functional effects on facial and dental structures (8). Thus, to reduce the risk of these complications, a thorough diagnosis and appropriate treatment strategies are of great importance.

Treatments of the cysts are mostly determined on the basis of their etiologies and localizations, each using different strategies to solve the problem and prevent recurrences or malignant growth (17). Surgical treatments for cysts, such as marsupialization and enucleation, were described in the early 1892s by Partsch and are still being used today (15,18). Enucleation and curettage appear to be appropriate treatments for most cases (19). Nevertheless, because many factors contribute to the treatments of odontogenic and non-odontogenic cysts (12,17), its epidemiology and treatment must be examined to better understand the nature of these lesions in different populations, especially in South Sulawesi, where proper documentation and sound statistics about public health are lacking. Hence, in this study, we aimed to investigate epidemiology and treatment of odontogenic and non-odontogenic cysts in South Sulawesi, Indonesia. We projected that the trends observed in our study would be comparable with those described in the recent literature and would be of diagnostic importance. Furthermore, this study could provide new and novel insights into the most prevalent oral pathologies encountered at one of the main national dental hospitals in Indonesia.

## Material and Methods

-Study population

This study was conducted retrospectively in patients treated at the the four different hospitals in Makassar between January 2011 and June 2017. Patients diagnosed as having odontogenic or non-odontogenic cysts and those treated for other cysts were included in the study. Patients who were not histopathologically diagnosed as having odontogenic or non-odontogenic cysts were excluded.

-Data collection

Data on the demographic variables that were collected for this study, such sex, age, and date of treatment, were noted in the data form. In addition, data on the clinical variables such as histopathological distribution based on the World Health Organization classification system (20), anatomical distribution, size of the cyst, and radiographic imaging findings were also noted. Moreover, we also investigated for the presence of any preoperative clinical signs and symptoms such as pain, numbness, swelling, pus, tooth mobility, trismus, speech problems, and swallowing problems. Treatment methods such as marsupialization, enucleation, curettage, tooth extraction, root canal treatment, and apicoectomy were documented. In addition, the type of reconstruction, duration of the operation, and complications during and/or after the operation were also recorded.

-Statistical analysis

The collected data were further processed using IBM SPSS Statistics 2.4. Descriptive statistics, including frequency, mean, minimum, maximum, and standard deviation (SD), were calculated. Further statistical analysis was performed using an independent T test and the Pearson chi-square test. A confidence interval of 95% (α = 0.05) was maintained for the analysis.

## Results

-Characteristics and distribution of the patients

**Figure 1:**
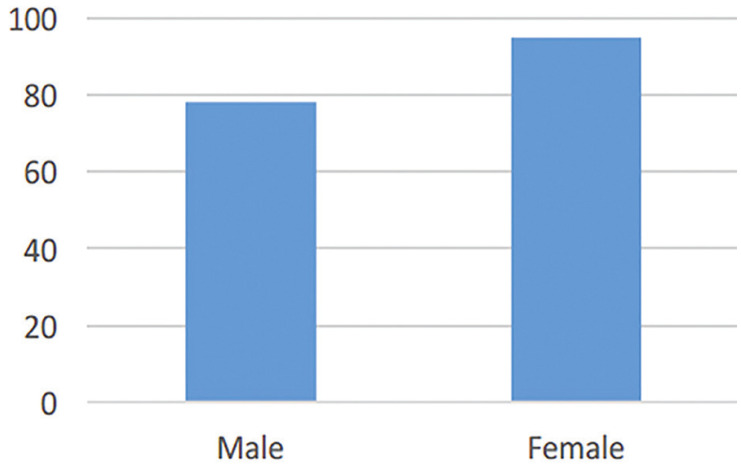
Gender distribution of all patients.

Data were collected for 173 patients treated for odontogenic and non-odontogenic cysts. These data were collected in four different hospitals in Makassar, but some data were missing from the medical records. Of the patients, 78 were male and 95 were female, with a male-to-female ratio of 1:1.21 (Fig. 1). The ages of 171 patients were known, ranging from 4 to 72 years, with a mean (SD) of 30.3 (17.2) years. The independent T test revealed no statistically significant difference between the age and sex groups (α = 0.05, *p* = 0.904).

**Figure 2:**
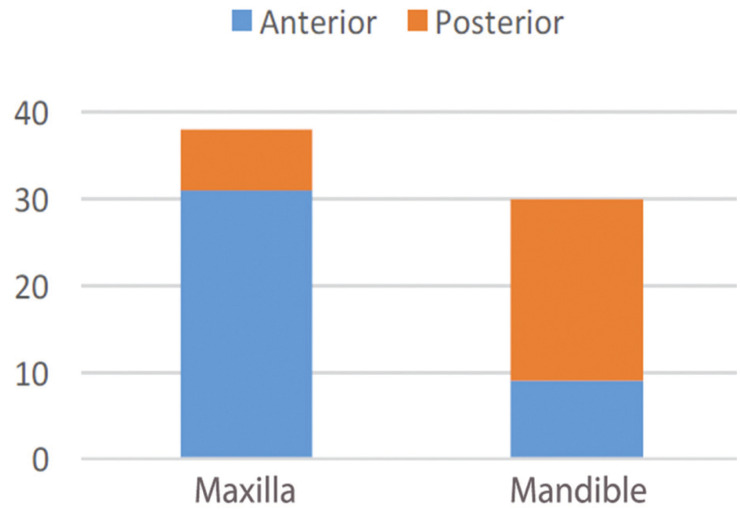
Cyst locations in the maxilla and mandible.

The exact locations of the suspected odontogenic cysts in 68 patients were identified. Of these cysts, 45.6% were in the anterior area of the maxilla; 30.9%, in the posterior area of the mandible; 13.2%, in the anterior area of the mandible; and 10.3%, in the posterior area of the maxilla (Fig. 2). Most of the suspected non-odontogenic cysts were in the mandible.

-Histological types and treatments

The histological type was known in only 60 patients (34.7%), and radiographic images were available for only 13 patients (7.5%). Forty-eight patients had odontogenic cysts, of whom 43.3% had radicular cysts, which were the most common, followed by dentigerous cysts (25.5%; Table 1). Four patients had pseudocysts, and 8 had non-odontogenic cysts, grouped as “others,” including mucoceles, ranulas, epidermoid cysts, globulomaxillary cysts, and retention cysts of the exocrine gland. The ratio of the odontogenic cysts to the non-odontogenic cysts was 6:1. The statistical analysis revealed no significant differences between the patients stratified according to sex and cyst type (α = 0.05, *p* = 0.485) and between those stratified according to sex and histological type of cyst (α = 0.05, *p* = 0.294).

**Table 1:**
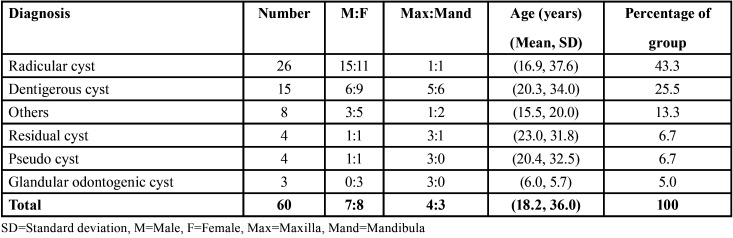
Histological distribution of cysts according to gender, anatomic site, and age.

**Figure 3:**
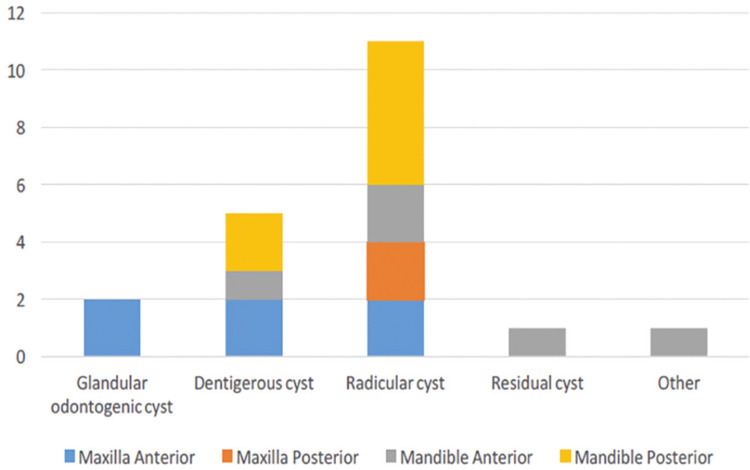
Specific anatomic site distribution for the histologically diagnosed cyst.

**Figure 4:**
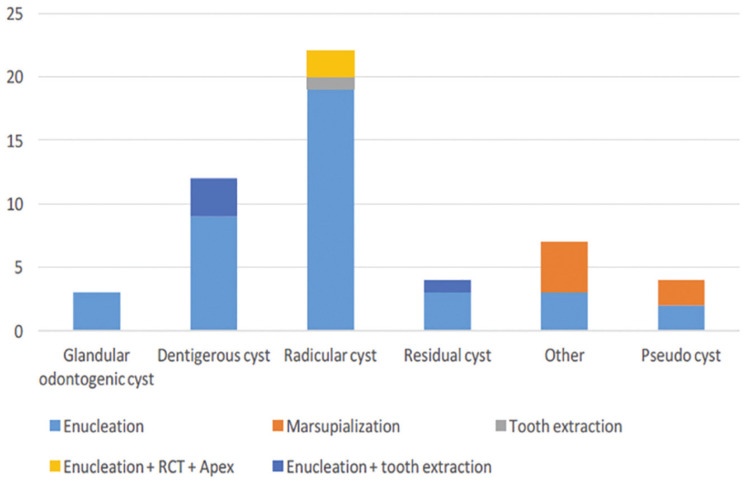
Treatment distribution among histopathological cyst.

On the basis of the histological analysis results, the exact anatomical site was known in 22 patients (Fig. 3). The radicular cysts were most frequent in the posterior area of the mandible, and the remaining cysts were distributed among the remaining sites in nearly equal proportions. No statistically significant difference was found between the patients stratified according to histological type and anatomical site of the cyst (α = 0.05, *p* = 0.277). Of the 60 patients who were diagnosed as having a cyst, 52 (86.7%) were treated and 8 (13.3%) were left untreated or had no documented treatment. The treatment distribution among the cysts is presented in Figure 4.

-Type of treatment and reconstruction

Of the 173 patients, only 154 were treated. In this study, different methods of treatment were used, of which enucleation was the most common (61.0%), followed by marsupialization (17.5%; Table 2). One patient who underwent marginal resection was suspected as having an odontogenic keratocyst (OKC; a differential diagnosis of ameloblastoma). No pathological result was available for this patient. The time to seeking medical assistance was known in 8 patients. The mean pre-presentation time was 12.8 months, ranging from 5 to 24 months. The tumor size was indicated in the medical records of 28 patients, three-dimensionally in 17 cases and two-dimensionally in 11 cases. For analysis, only the largest dimension was used. The mean (SD) size was 32.4 (22.1) mm, and the sizes ranged from 5 to 80 mm.

**Table 2:**
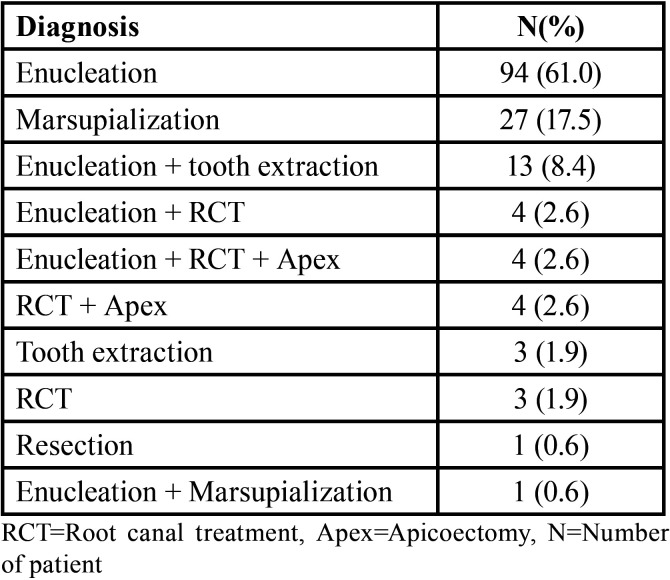
Different treatment methods used.

In 53 patients (51.8%), reconstruction primarily involved placing an obturator postoperatively and more frequently after removal of the radicular and dentigerous cysts in 50.9% and 39.6% of the cases, respectively. One titanium plate reconstruction was performed immediately after segmental resection of the mandible. None of the patients who had non-odontogenic cysts underwent any form of reconstruction.

-Complications

Preoperative clinical symptoms occurred in 33 patients (19.1%), of which the most common were pus and swelling (Table 3). Surgical reports were not available for all these patients, which made the surgical complications difficult to analyze preoperatively. Postoperative complications, namely fever, pain, and swelling, all occurred in only 3 patients (1.7%). All three patients experienced.

**Table 3:**
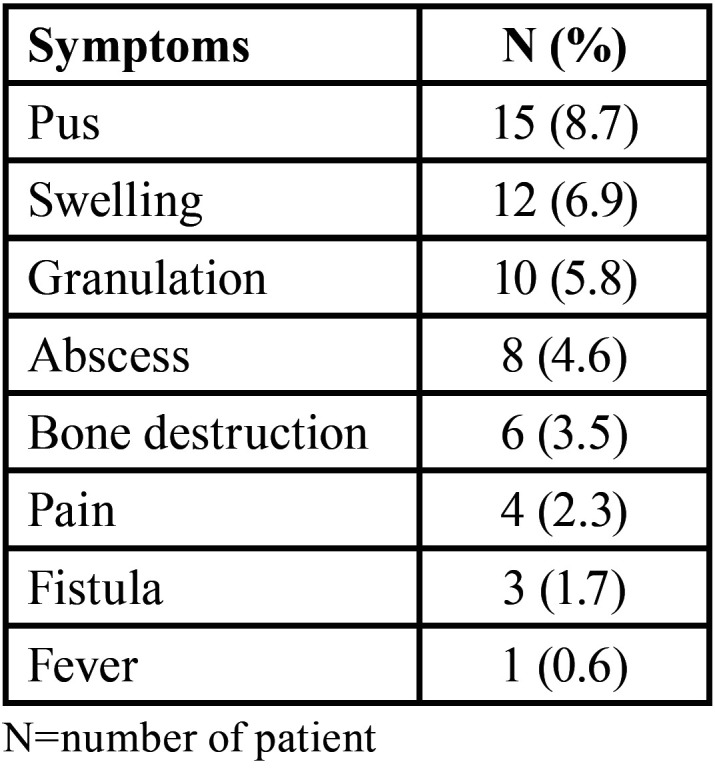
Pre-operative clinical symptoms.

-Follow-up and recurrence

Follow-up was performed for 126 patients (72.8%). Of these patients, 118 (93.7%) underwent follow-up within 1 year; and 8 (6.3%), within 2 years. The mean (SD) follow-up frequency was 3.6 (2.3) times, with a range of 1 to 12 times. Only 5 patients (3.2%) experienced a recurrence within 1 year, of whom 4 had a recurrence after enucleation of a radicular cyst. After cyst removal, all 4 patients received an obturator. One patient had a recurrence after marsupialization of the mucocele in the labia inferior. Radiographic images were obtained for all the patients but were only available for 22 patients.

## Discussion

Epidemiological studies are known as the basis for formulating health policies (10,13). Knowledge of the most common diseases, including their etiologies, risk factors, prevention, and treatments, is also useful (10). In the present study, we aimed to investigate the epidemiologies and treatments of odontogenic and non-odontogenic cysts over a 6-year period in South Sulawesi, Indonesia. All data were collected from several hospitals in Makassar, the capital city of South Sulawesi, Indonesia. Makassar is a dynamically developing city where medical facilities are constantly improving. Initially, we hoped to investigate the epidemiologies and treatments of odontogenic and non-odontogenic cysts over a 10-year period. Unfortunately, well-documented data in Makassar were available only up to 6 years retrospectively. Before that time, all records were only documented in patient files. As the operations were not logged, reviewing all the handwritten files was difficult.

To establish and characterize the real incidence of OMF diseases, studies with histopathological data are of great importance (14). Theoretically, clinicians should provide all information related to the clinical and radiological findings to pathologists to infer an overall impression (6). In the present study, data were collected for 173 patients over a period of 6 years. All data collected from the logbook were submitted for histopathological analysis, but histopathological results were only available for 60 patients. This clearly indicates that not all histopathological forms were completely filled out with all the parameters for each specimen. However, whether the analysis was performed for all the patients or whether only proper documentation was lacking was unclear. However, the prevalence rates of the histopathological types reported in this study should not be confused with the real prevalence rates of odontogenic and non-odontogenic cysts in Makassar because many lesions were diagnosed on the basis of clinical and radiological information rather than histopathologically. This was due to the experience and general knowledge of practitioners, which resulted in the exclusion of some patients from the analysis.

Generally, odontogenic and non-odontogenic cysts are mostly discovered incidentally during regular dental checkup and thus can be undetected for several years if no dental imaging is performed by the clinician or no clinical symptoms that suggest any pathology are present (16,21). Therefore, patients must visit their dentists on a regular basis for checkup and follow-up to avoid any further destruction of the oral structures. In developing countries such as Indonesia, patients often wait until the lesion has affected their oral health negatively before seeking medical care. In this study, one patient had complaints for nearly 24 months before seeking medical care. The patient complied with the treatment and showed a good clinical course during follow-up. However, within a few months after the treatment, the lesion recurred with a differential diagnosis of ameloblastoma. Fortunately, the lesion was histopathologically categorized as a benign cyst and was later treated and managed successfully, which led to a positive prognosis.

Regarding the patient distribution in this study, we found that cysts were slightly predominant in females but relatively equal for both sexes. This result agrees with the finding of Grossman *et al*. that the sex distribution of patients was 49.26% females and 48.78% males (4). In another study, Johnson *et al*. found that the sex distributions of odontogenic and non-odontogenic cysts both showed male predominance but with a slight difference (1.2:1) (6). With regard to patient age at diagnosis, in this study, we discovered that the cysts appeared most likely between the second and third decades of life, similarly to those reported in the literature, which were found between the first and eighth decades of life (4,22,23). The preferred location of the cysts in our study was the anterior maxilla, followed by the posterior mandible. Our result is in line with the previous report of jaw cysts mostly located in the maxilla (2701 cases, 53.09%) (24). Emphasizing this result, another study also reported that jaw cysts were more prevalent in the anterior region of the maxilla, followed by the molar area of the mandible (25). This is not a surprising finding considering the fact that the third lower molar and upper canine are the most commonly impacted teeth, which can trigger the development of dentigerous cysts (4,7).

In the present study, pathological analysis revealed that of all the cysts, 43.3% were of the radicular type and 25.5% were of the dentigerous type. This is in accordance with the previous studies that identified radicular and dentigerous cysts as the most prevalent types (26-29). Furthermore, in our study, we found only one case each of suspected OKC, nasolabial cyst, and nasopalatine cyst, but no pathological analysis records were available for these cases. Even if the case of OKC, which occurred in the posterior mandible, was included in the study, the prevalence rate would still be very low as compared with those reported in a recent study (24). In a systematic review, Jones *et al*. found that the prevalence of OKC was as much as 11.7% (7), which is different from the result of our study. This may be due to the fact that the case of suspected OKC in the present study was followed up for only 1 year, whereas in the existing literature, cases of OKC usually recur after a mean of 5 years postoperatively (30).

The appropriate treatment methods for cystic lesions introduced by Partsch are enucleation for small cysts (diameter, <2.5 cm) and marsupialization for large cysts (diameter, ≥2.5 cm) (15,18). A marsupialization followed by cyst drainage will reduce the pressure in the cyst and therefore would reduce the size of the cyst, making enucleation in one piece less difficult and reducing the risk of recurrence (15,18). However, all the odontogenic cysts in this study, including those >2.5 cm were treated with enucleation only. Five specimens >2.5 cm in size that were sent to the laboratory for histopathological analysis after enucleation were not intact, but the patients had no recurrence. In this study, reconstruction with an obturator was performed for 51.8% of the cysts, which were all followed up. The recurrence rate of the radicular cysts after enucleation was 75%. Similarly, Grossman *et al*. also reported recurrences of radicular cysts and suggested that these were partially due to inadequate treatment of the primary lesion (4).

## Conclusions

This study confirms that the radicular cyst is the most frequent cyst in South Sulawesi, Indonesia, followed by the dentigerous cyst. The incidence of odontogenic and non-odontogenic cysts widely varies, with some exhibiting a predilection for specific ages and sites and a specific sex. Therefore, the importance of visiting a dentist regularly and a dental practitioner for radiographic imaging must be emphasized to patients so that pathological conditions can be intercepted earlier. Furthermore, a strict protocol is required for proper documentation to obtain the complete information of each patient for precise treatment, future studies, and references.
